# Mutation analysis of the LCE3B/LCE3C genes in Psoriasis

**DOI:** 10.1186/1471-2350-11-45

**Published:** 2010-03-23

**Authors:** Eliecer Coto, Jorge Santos-Juanes, Pablo Coto-Segura, Marta Díaz, Javier Soto, Rubén Queiro, Victoria Alvarez

**Affiliations:** 1 Genética Molecular, Hospital Universitario Central Asturias-Servicio de Salud del Principado de Asturias, Oviedo, Spain; 2 Unidad Dermatología II, Hospital Universitario Central Asturias-Servicio de Salud del Principado de Asturias, Oviedo, Spain; 3 Servicio de Reumatología, Hospital Universitario Central Asturias-Servicio de Salud del Principado de Asturias, Oviedo, Spain; 4 Department of Medicine, University of Oviedo, Oviedo, Spain

## Abstract

**Background:**

An association between a common deletion comprising the late cornified envelope LCE3B and LCE3C genes (LCE3C_LCE3B-del) and Psoriasis (Ps) has been reported. The expression of these LCE genes was induced after skin barrier disruption and was also strong in psoriatic lesions. The damage to the skin barrier could trigger an epidermal response that includes the expression of genes involved in the formation of skin barrier.

**Methods:**

We determined the LCE3C_LCE3B-del genotype in 405 Ps patients and 400 healthy controls from a Northern Spain region (Asturias). These patients and controls were also genotyped for the rs4112788 single nucleotide polymorphism, in strong linkage disequilibrium with the LCE3C_B cluster. The LCE3B and LCE3C gene variant was determined in the patients through SSCA, DHPLC, and direct sequencing.

**Results:**

Allele and genotype frequencies did not differ between patients and controls for the rs4112788 and LCE3C_LCE3B-del polymorphisms. However, del/del homozygotes were significantly higher among patients with chronic plaque type Ps who did not develop arthritis (p = 0.03; OR = 1.4; 95%CI = 1.03-1.92). The analysis of the coding sequence of LCE3B and LCE3C in the patients who had at least one copy of this showed that only one patient has a no previously reported LCE3B variant (R68C).

**Conclusion:**

Our work suggested that homozygosity for a common LCE3C_LCE3B deletion contributes to the risk of developing chronic plaque type Ps without psoriatic arthritis. Our work confirmed previous reports that described an association of this marker with only skin manifestations, and supported the concept of different genetic risk factors contributing to skin and joint disease.

## Background

Psoriasis (Ps) is a chronic hyperproliferative inflammatory disease of the skin that affects approximately 2% of individuals [1]. Ps is characterized by an abnormal keratinocyte proliferation and differentiation, and the infiltration of immunocompetent cells in the epidermis and dermis [2]. Psoriatic arthritis (PsA) is a chronic inflammatory joint disease which occurs in 7-42% of Ps patients [3]. Ps susceptibility involves environmental and genetic factors [4]. The genetic component is partly explained by its association to the HLA-Cw6 allele, that defines the PSOR1 locus on chromosome 6p21 [5,6]. Genome-wide association studies (GWA) and family-based studies have identified other Ps associated genes, and some of them encode proteins involved in the immune response and expressed in keratinocytes and T cells [7-10].

Copy number variants (CNVs) are an important source of genetic variability, and contribute to the susceptibility to several diseases [11]. A candidate gene approach identified a higher copy number of the beta-defensin cluster as a risk factor for Ps, and a recent genome-wide CNV analysis identified the association of Ps with a deletion comprising the *LCE3B *and *LCE3C *genes (LCE3C_LCE3B-del) [9,12]. These genes encode members of the late cornified envelope (LCE), and are in the region that contains the PSORS4 locus on chromosome 1q21 [13,14]. The mRNA for several LCE3 genes was absent in normal skin, but its expression was induced after skin barrier disruption and was also strong in psoriatic lesions [9]. In this way, the damage to the skin barrier could trigger an epidermal response that includes the expression of genes involved in the formation of skin barrier, such as those in the LCE cluster. The impaired response in individuals homozygous for the LCE3C_LCE3B-del could explain the higher risk for Ps conferred by this genotype. The reported lack of association between the LCE3C_LCE3B-del and psoriatic arthritis suggested that this was a risk factor for only skin disease, supporting the concept that different genetic factors contributed to skin disease and joint manifestations [15,16].

Here, we report the results of a case-control study for the association between the LCE3C_LCE3B-del and Ps. In addition, we searched for DNA variants in the coding sequences of *LCE3C *and *LC3B *in Ps patients.

## Methods

### Patients and controls

All the patients and controls were Spanish Caucasians from the region of Asturias (Northern Spain, total population 1 million). A total of 405 non-related patients with Ps (mean age 47 ± 16 years; 54% men) were recruited by Dermatologists from Hospital Universitario Central Asturias (HUCA) and Hospital A. Buylla-Mieres. Ps was diagnosed based on clinical findings, and the Psoriasis Area and Severity Index (PASI) was determined. Patients who were diagnosed with arthritis prior to Ps were not included in the study. The disease was considered as severe in patients with a PASI score = 10 [17]. Patients were considered to have "*early onset*" psoriasis if the onset of the disease was at any age ≤ 40 years, and "*late onset*" psoriasis if the onset was > 40 years. Patients were considered to have "*familial*" psoriasis if they had at least one first- or second-degree relative affected by the condition. Patients with PsA were assessed by a rheumatologist according to the criteria of Moll and Wright [18]. All the patients were followed for >2 years after psoriasis was diagnosed (mean follow-up, 17 ± 14 years). Table 1 summarizes the main characteristics of the patients.

**Table 1:** Main characteristics of the 405 patients with Psoriasis.

Gender (male/female)	210 (54%)/195(46%)
Mean age (years ± SD)*	47 ± 16
Type of psoriasis	
Vulgaris	324 (80%)
Palmoplantar	49 (12%)
Guttate	16 (4%)
other	16 (4%)
	
Cw6+	158 (39%)
	
Affected relatives (familial Ps)	219 (54%)
	
Early-onset psoriasis^#^	243 (60%)
Mean age (years ± SD)*	34 ± 14
Cw6+	124 (51%)
Late-onset psoriasis^#^	162 (40%)
Mean age (years ± SD)*	59 ± 9
Cw6+	34 (21%)
Non-severe Ps (PASI < 10)*	243 (60%)
	
Severe Ps (PASI ≥ 10)*	162 (40%)
	
Arthritis	89 (22%)
	
Non-Arthritis	316 (78%)

^#^Early onset psoriasis, age ≤40 years; Late onset psoriasis, age>40 years.

* SD, standard deviation; PASI, Psoriasis Area and Severity Index.

The control group comprised 400 non-related healthy individuals (mean age 49 ± 16 years; range 18-85 years; 55% men) recruited through the Dermatology Department (Hospital staff and healthy spouses of patients) and the HUCA-Blood Bank. The study was approved by the Ethical Committee of HUCA, and all the individuals gave their informed consent to participate.

### LCE3C_LCE3B-del genotyping

We determined the LCE3C_LCE3B-del genotype following a three-primer polymerase chain reaction (PCR) assay, as reported [9]. Genomic DNA from patients and controls was PCR-amplified in a single tube containing each of three primers: LCE3F (forward), LCE3CR (reverse), and LCE3CR2D (reverse). Ten μl of each reaction were electrophoresed on 2% agarose gels, and the non-deletion allele visualized as a fragment of 240 bp (LCE3CF-LCE3CR product), and the deletion as a fragment of 199 bp (LCE3CF-LCE3CR2D product).

### SNP rs412788 genotyping

SNP rs4112788 (T/C) was genotyped in patients and controls through restriction enzyme digestion of a PCR fragment generated with primers Fwd: 5'GTAGAGACTAACCATATAACATGTGG (forward) and Rvs: 5'GAAAACCTTTAGACTACAATTAAAAGC (reverse) (PCR annealing, 55°C). The last nucleotide in Rvs has a mismatch to create a site for the restriction enzyme *MspI *on PCR fragments with the C allele. After digestion with this enzyme and electrophoresis on 3% agarose gels, alleles were visualized as bands of 215 bp (T) or 190 + 25 bp (C).

A total of 150 patients and 150 controls (representing at least 50 of the three PCR-RFLP genotypes) were also genotyped with a custom Taqman assay for SNP rs4112788 (assay id C_31910050_10) in a real time PCR Step One (ABI).

To determine the accuracy of the two genotyping methods we sequenced 20 individuals with each of the three genotypes. A 330 bp fragment was amplified with primers Fwd (see above) and 5' GACTCTCCAAGGGACATTTTTTGT (reverse) (annealing at 56°C), and both strands were sequenced.

### LCE3B/3C mutation screening

We amplified the *LCE3B *and *LCE3C *coding sequences in the patients with primers designated from the reference sequences http://www.ensembl.org; ENSG00000187238 for *LCE3B*; ENSG00000187238 for *LCE3C*). The full transcripts encode proteins of 96 amino acids are contained in single exons. The *LCE3B *fragment was 422 bp long and was amplified with primers GGGCTTCATAAAACCATTTGTAGAG (forward) and TTTCCTCTAAAGTCGCTTGTCTCA (reverse) (annealing, 63°C). The *LCE3C *was a 448 bp fragment amplified with primers GGTCTGAGGGTTCTGTGCTCA (forward) and TCTGGAAAAGCATGCATCAGG (reverse) (annealing, 62°C).

To search for DNA variants, a total of 45 *LCE3B *and *LCE3C *fragments (corresponding to 15 ins/ins patients and 15 ins/del patients) were sequenced. PCR fragments from all the patients were also subjected to single strand conformation analysis (SSCA), and fragments with atypical SSCA electrophoretic patterns were sequenced to characterize the nucleotide changes [19]. These PCR fragments were also subjected to Denaturing High Performance Liquid Chromatography (DHPLC) in a Varian Helix System and with a linear binary gradient created with buffers Varian Helix A (triethylammonium amine - TEAA) and B (TEAA+25% acetonitrile; http://www.varianinc.org), and the nucleotide changes responsible for the different elution profiles were identified by sequencing the corresponding PCR fragments. The DHPLC elution temperatures and buffer gradients for the PCR fragments were calculated with the DHPLC Melt Program http://insertion.stanford.edu/melt.html, and are available upon request to the corresponding author.

### Statistical analysis

Data management and statistical analysis were carried out using SPSS for windows (release 15.0; SPSS, Inc). Differences of the allele frequencies between the groups were assessed using the χ^2 ^test. Odds ratios (OR) and their 95% confidence intervals (CI) values were also calculated. The Student's *t *test was used to compare the quantitative data between the groups. A p < 0.05 was considered as statistically significant. The power of the study for each frequency comparison was calculated online http://statpages.org/proppowr.html.

## Results

### LCE3C_3B del frequencies

**Figure 1:**
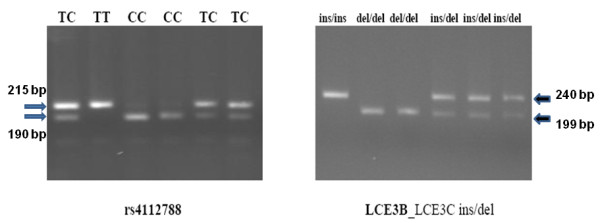
**PCR-RFLP genotypes of rs4112788 (right) and LCE3B_3C ins/del genotypes (right)**. The rs4112788 alleles were visualized as bands of 215 bp (T) and 190 + 25 bp (C). The LCE3B_3C insertion and deletion alleles were visualized as PCR fragments of 240 bp and 199 bp.

We determined the genotype for the common LCE3C_LCE3B CNV in 405 Ps patients and 400 controls (Figure 1). Genotype and allele frequencies for the LCE3C_LCE3B-del in patients and controls are summarized in table 2. The observed genotype frequencies did not differ from those expected under the Hardy-Weinberg equilibrium. We did not find significant differences for the genotype and allele frequencies between the controls and total patients. The del/del frequency was higher among patients with Ps vulgaris (n = 324) compared to controls (42% vs. 36%) although the difference did not reach statistical significance (p = 0.08; OR = 1.30, 95CI = 0.96-1.76).

**Table 2:** Genotype and allele frequencies for the LCE3C_LCE3B-del (parentheses indicate frequencies).

	LCE3 genotypes			Alleles	
	**II**	**ID**	**DD**	**D**	**I**

Controls (n = 400)	60 (15)	196 (51)	144 (36)	493 (0.61)	307 (0.39)

Total patients (n = 405)	52 (13)	204 (50)	149 (37)	502 (0.62)	308 (0.38)

Psor. Vulgaris (n = 324)	30 (9)	157 (49)	137 (42)	431 (0.67)	217 (0.33)

Familial Ps (n = 221)	32 (14)	113 (51)	76 (34)	265 (0.60)	177 (0.40)
Sporadic Ps (n = 184)	20 (11)	91 (50)	73 (39)	237 (0.64)	131 (036)

Early onset Ps (n = 243)	32 (13)	124 (51)	87 (36)	298 (0.61)	188 (0.39)
Late onset Ps (n = 162)	20 (12)	77 (48)	65 (40)	207 (0.64)	117 (0.36)

Non Severe Ps (n = 243)	28 (11)	126 (52)	89 (37)	304 (0.63)	182 (0.37)

Severe Ps (n = 162)	24 (15)	78 (48)	60 (37)	198 (0.61)	126 (0.39)

Arthritis (n = 83)	8 (10)	49 (59)	26 (31)	101 (0.61)	65 (0.39)

Non arthritis (n = 322)	44 (14)	155 (45)	123 (41)	401 (0.62)	243 (0.38)

Cw6 positive (n = 158)	11 (8)	81 (51)	66 (42)	213 (0.67)	103 (0.33)

Cw6 negative (n = 247)	40 (16)	123 (50)	84 (34)	291 (0.59)	203 (0.41)

Psor. Vulgaris Non arhritis (n = 274)	25 (9)	128 (47)	121 (44)	370 (0.68)	178 (0.32)

Because a previous study reported a negative association between the LCE3C_LCE3B-del and psoriatic arthritis, we compared the genotype frequencies between controls and patients who did not develop arthritis (n = 274). The frequency of del/del was significantly higher in this group compared to controls (44% vs. 36%; p = 0.03; OR = 1.4 (1.03-1.92). However, the number of patients/controls was insufficient to reach a power of 80. At a significance level of p = 0.05, with del/del frequencies of 0.36 in controls and 0.44 in patients and a control/patient ratio of 1.46, a total of 1,154 controls and 938 patients should be genotyped to reach a power of 80 (% chance of detecting).

### SNP rs4112788 genotype frequencies

**Figure 2:**
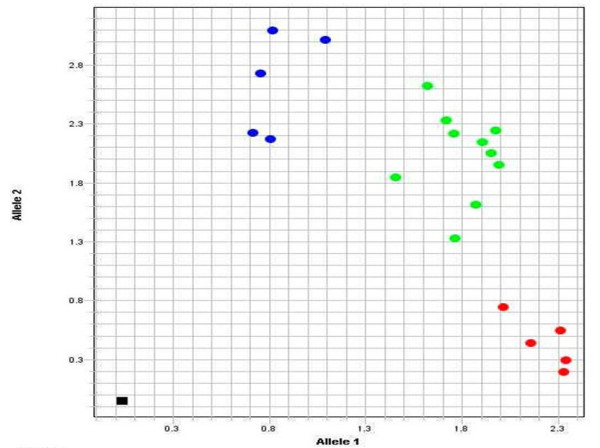
**Taqman genotyping of SNP rs4112788 in 20 individuals, 5 CC (blue dots), 10 CT (green dots), and 5 TT (red dots)**. The black spot in the down-left was the negative control.

SNP rs4112788 in the *LCE3D *gene was genotyped in all the patients and controls through PCR-RFLP (Figure 1). This polymorphism maps approximately 4.5 Kb centromeric to the LCE3C_LCE3B-del, and allele C has been reported in almost complete linkage disequilibrium (LD) with the deletion. In agreement with this, all our patients and controls who were homozygous for the deletion were also rs4112788 CC, while all the non-deletion homozygotes were also TT. All but two ins/del individuals were also CT (one patient and one control were CC). To determine the accuracy of the PCR-RFLP method, we first sequenced 60 individuals, 20 each of the three genotypes. We confirmed the genotype in all the 60 samples, and we also found a complete disequilibrium between rs4112788 and rs4112787 (T/C), a SNP located 49 bp 3' to rs4112788. A total of 100 patients and 100 controls were also genotyped with a custom Taqman assay (Figure 2). We only found one discrepancy between the RFLP and Taqman methods: one patient genotyped as TC with the PCR-RFLP method was CC according to the Taqman assay, and sequencing confirmed the RFLP genotype. Thus, the accuracy of our PCR-RFLP genotyping method should be almost complete.

### Mutation analysis of *LCE3B *and *LCE3C*

**Figure 3:**
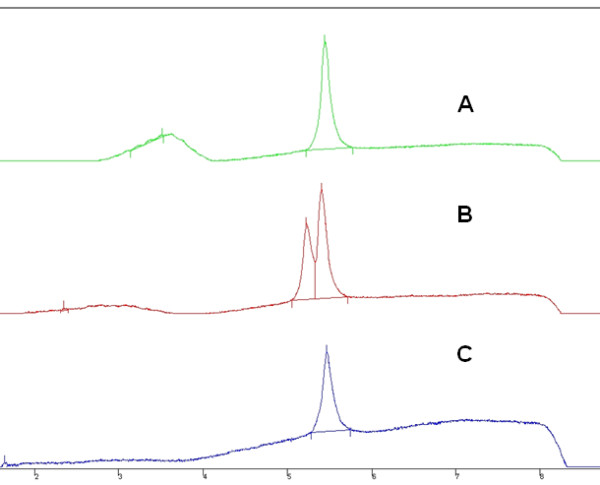
**DHPLC profiles of three *LCE3B*-PCR fragments**. The normal sequences gave a single elution peak, while the fragment with a nucleotide change eluted as a double peak (B). Fragments were eluted with the universal gradient of Varian Helix buffers A and B http://www.varianinc.org/, and at a temperature of 60°C.

We searched for DNA variants in the *LCE3B *and *LCE3C *coding sequences in the patients. After electrophoresis of the PCRs, a band was only visible in the 353 patients with at least one copy of LCE3C_LCE3B. This result confirmed the absence of the two LCE3C_LCE3B copies in the 149 patients who were genotyped as homozygotes for the deletion. The sequencing of 45 chromosomes (15 ins/ins and 15 ins/del patients) showed no nucleotide variants in the two genes. SSCA and DHPLC indicated the absence of nucleotide changes in all the *LCE3C *PCR-fragments. Only one patient showed an *LCE3B *heterogeneous SSCA/DHPLC pattern (Figure 3). After sequencing, we found that this individual (a 28 year-old patient with chronic plaque type Ps without arthritis and heterozygous for the deletion) had a missense change: R68C (CGC>TGC). We did not find this nucleotide change in the 400 controls (genotyped through SSCA).

## Discussion

SNP rs4112788 and the LCE3C_LCE3B-del have been associated with Ps [9,17,20]. In their analysis of 1,425 patients and 1,406 controls from four different populations, De Cid et al. found a significantly higher frequency of del/del homozygotes among the patients (total del/del frequency, 0.45 in patients vs. 0.34 in controls; OR = 2.04, 95% CI = 1.59-2.62, p = 1.71e-08). This was further confirmed by Hüffmeier et al. in individuals of German origin [15]. We did not find this difference when all the Ps patients were compared to controls. However, in agreement with these authors the del/del genotype was more frequent among patients with psoriasis vulgaris, although our OR was lower and did not reach statistical significance, probably due to the limited sample size [9,15].

Huffmeier et al. reported a lack of association between the LCE3C_LCE3B-del and PsA among German patients [16]. We found a lower frequency of del/del among the patients who developed arthritis, compared to patients with only skin disease. Moreover, when patients with Ps vulgaris and without joint manifestations were compared to controls, we found a significantly higher frequency of the del/del genotype. This confirmed the reported association of this marker with only skin disease [15,16].

The association between the LCE3C_LCE3B-del and Ps vulgaris was weaker in our population compared to the reported by others. This is illustrated by the fact that for an OR of 1.46 and a p = 0.05 at least 1,154 patients (Ps vulgaris without joint disease) and 938 controls should be genotyped to reach a power of 80. This discrepancy with other studies could be partly attributed to differences in the main characteristics of patients and controls. De Cid et al. analysed a total of 175 patients and 382 healthy controls from a different Spanish region [9]. The deletion allele had almost the same frequency in their patients (0.64) and ours (0.62), and the significant risk for Ps was due to a lower frequency of the deletion among their controls (0.55 vs. 0.61). The lower association in our study could be thus attributed to differences in the criteria followed by the recruitment of these controls. However, this is unlikely because controls in the two studies were healthy individuals from the general population and with similar mean age. Mistyping of the LCE3C_LCE3B alleles in some of our patients and controls could also explain the results, but this was also unlikely because we found an identical degree of association with rs4112788, a SNP in almost complete LD with the LCE3C_LCE3B-del. Moreover, the genotype frequencies in our patients and controls were in Hardy-Weinberg equilibrium, suggesting they were representative of the general population.

Differences in the main clinical characteristics of the patients could also explain the lack of association in our study. Compared to others, our work included patients with a higher mean age, and a lower proportion of familial and severe cases. This could explain the low frequency of Cw6+ among our cases, a marker reported to be more common among severe early-onset Ps [21]. The frequency of this marker was also more frequent among our patients with familial and early-onset Ps. In this way, the effect of the deletion allele on Ps-risk could be higher in patients with an early onset, familial, and severe disease, and this could result in a reduced frequency of LCE3B_LCE3C-del among our cases.

Finally, the analysis of *LCE3B *and *LCE3C *coding sequences in cases with at least one copy of these genes showed that only one patient had a missense change at a conserved *LCE3B *amino acid (R68>C). This was not found in the controls. SSCA and DHPLC are indirect techniques to detect nucleotide changes in PCR fragments and, although they have a low rate for false negatives, we cannot exclude that some of the patients harbour non identified mutations.

## Conclusions

Our work suggested that individuals homozygous for the LCE3C_LCE3B-del are at risk of developing Ps vulgaris without arthritis manifestation. This confirmed the recently reported association between this marker and only skin psoriasis, reinforcing the concept of Ps as a deregulation in the wound response programs.

## Competing interests

The authors declare that they have no competing interests.

## Authors' contributions

EC, JSJ, and VA designated the work, analyzed the results, and wrote the manuscript. JSJ, PCS, JS, and RQ recruited the patients and controls and obtained the clinical, analytical and anthropometric data. EC, MD, and VA performed the genetic studies. All the authors revised and approved the final version of the manuscript.

## Pre-publication history

The pre-publication history for this paper can be accessed here:

http://www.biomedcentral.com/1471-2350/11/45/prepub
